# Iridophoroma associated with the Lemon Frost colour morph of the leopard gecko (*Eublepharis macularius*)

**DOI:** 10.1038/s41598-020-62828-9

**Published:** 2020-03-31

**Authors:** Paweł Szydłowski, Jan Paweł Madej, Magdalena Duda, Janusz A. Madej, Agnieszka Sikorska-Kopyłowicz, Anna Chełmońska-Soyta, Lucyna Ilnicka, Przemysław Duda

**Affiliations:** 1 Department of Immunology, Pathophysiology and Veterinary Preventive Medicine, Faculty of Veterinary Medicine, Wroclaw University of Environmental and Life Sciences, Norwida 31, Wroclaw, 50-375 Poland; 2 Department of Histology and Embryology, Faculty of Veterinary Medicine, Wroclaw University of Environmental and Life Sciences, Norwida 25, Wroclaw, 50-375 Poland; 3 Department of Internal Diseases and Clinic of Diseases of Horses, Dogs and Cats, Faculty of Veterinary Medicine, Wroclaw University of Environmental and Life Sciences, pl. Grunwaldzki 47, Wroclaw, 50-366 Poland; 4 Department of Pathology, Faculty of Veterinary Medicine, Wroclaw University of Environmental and Life Sciences, Norwida 31, Wroclaw, 50-375 Poland; 5 Department of Epizootiology and Clinic of Bird and Exotic Animals, Faculty of Veterinary Medicine, Wroclaw University of Environmental and Life Sciences, pl. Grunwaldzki 45, Wroclaw, 50-366 Poland; 6 Department of Molecular Physiology and Neurobiology, University of Wroclaw, Sienkiewicza 21, Wroclaw, 50-335 Poland

**Keywords:** Skin cancer, Herpetology

## Abstract

The Lemon Frost is a new colour morph of the leopard gecko, which emerged in ca. 2015 as a result of selective breeding and spontaneous mutation. According to multiple breeders observation of Lemon Frost inbreeding with wild-type leopard geckos, Lemon Frost seems to be a codominant trait. Additionally breeders observed another, presumably associated trait - tumour-like skin lesions. Three private-owned Lemon Frost morph leopard geckos with tumour-like skin lesions were admitted to our clinic for examination, which included histopathology, X-ray and ultrasonography. The histopathological investigation of the biopsies indicated malignant iridophoroma; however, no changes were observed in diagnostic imaging. This research is the first report of clinical and histopathological findings of iridophoroma in leopard geckos.

## Introduction

The leopard gecko (*Eublepharis macularius*, Blyth 1845) is a nocturnal species naturally found in Afghanistan, Pakistan, India, Iran and Nepal^1,2^. Additionally, the leopard gecko is one of the most popular breeding species and has been kept by private owners for over thirty years. As the result of long-term breeding programmes, about one hundred colour morphs have come into existence to date.

**Figure 1:**
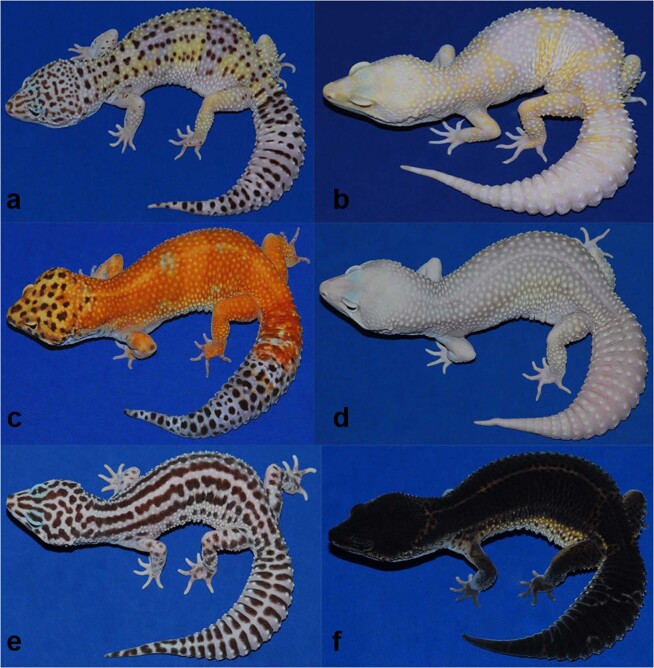
Examples of leopard gecko colour morphs. Wild-type (**a**) with a normal arrangement of dots (melanophores), yellow colour (xathophores). The Tremper albino leopard gecko (**b**) with colourless dots and stripes (lack of melanin in melanophores). Example of the hypomelanistic colour morph (**c**) – a lack of dots on the dorsal part of body. Lack of any black coloured dots (amelanism) and the yellow pigmentation (axanthism) in a non-albino colour morph called “Blizzard” (**d**). Axantic (lack of yellow pigmentation) colour morph (**e**). Hipermelanistic colour morph (**f**). Photo by Steve Sykes - Geckos Etc.

Reptile skin colouration depends on a distribution and presence of the chromatophores, which include the melanophores, the xanthophores, the erythrophores and the iridophores^3–5^. These cells originate from a differentiation of neural crest stem cells^5^. Melanophores are dark brown cells containing melanosomes filled with melanin. Xanthophores and erythrophores contain vesicle structures with carotenoid or pteridine pigments ranging from yellow to red^4^. Iridophores are light-reflecting cells containing light-reflecting platelets made up of crystalline guanine, adenine, hypoxanthine or uric acid inclusions^6^; the ultrastructure and arrangement results in white, blue to red skin colouration^7^. The wild-type adult leopard gecko has a skin pigmentation pattern made up of a yellow-and-black-spotted dorsal part, a greyish tail with white transversal stripes and black dots, a yellowish head with black dots and a white/light cream ventral part of the body^8^. On account of different types of chromatophores and the morphology of leopard gecko colour morphs, a few categories exist to describe these morphs. The basic colour morphs of leopard gecko can be found in Fig. 1. There are simple colour morphs as well as combinations of them. Colour morphs describe the presence and distribution of melanophores and xathophores. In short: hypo- and hyperxanthic morphs (respectively less or more intense yellow pigmentation, e.g. Tangerine, High Yellow), axanthic (lack of yellow pigmentation, e.g. Mack Super Snow, Blizzard), hypomelanistic (less intense black-brown pigmentation or less numerous dorsal dots and spots) and three different albino strains (Bell, Las Vegas and Tremper albino). All names of colour morphs are derived from the breeder. Hence, there are no formal and scientific rules to describe currently existing colour morphs of the leopard geckos. Selective breeding has led to combinations of existing colour morphs and a development of new ones. One of them, the Lemon Frost, is characterized by an increased white body colouration and a brightening of the yellow/orange areas of the body. In ca. 2015 new colour morph called Lemon Frost, emerged as a result of selective breeding. Since that time the breeders observed that large number of these geckos were affected by numerous tumour-like skin lesions.

The genetics of the leopard geckos are well developed. It is known that they have 38 chromosomes (2n), their genome size is 4.91 picograms (2c) and it contains 43.66% of GC nucleotides^9^. Additionally, a genome with high coverage sequenced what revealed that leopard geckos have 24,755 protein-coding genes^10^. Despite these facts the genetic character of the colour morphs are still unclear and the only information is based on private breeders’ observations. In this case, according to breeders contest, the Lemon Frost phenotype seems to be a codominant trait. Apart from their unique morphology, the Lemon Frost colour morph is associated with nodular skin lesions.

In veterinary practice, reptile neoplasms affecting any kind of tissue or organ are not frequently observed^11^, and skin tumours seem to be particularly rare. The frequency of chromatophoromas in reptiles is estimated at 14.5%, and melanophoromas (11.2%) are more often encountered than iridophoromas (3.3%)^12^. Melanophoromas have been reported in several cases^11–13^. Iridophoromas have been found in a few cases, e.g in *Pituophis melanoleucus* and *Morelia viridis*^14^. In lizards iridophoromas were reported and well-described in *Pogona vitticeps*^12^. There is much evidence that iridophoromas in reptiles may be either benign or malignant. Benign iridophoromas have been reported in veiled chameleon (*Chamaeleo calyptratus*), a bearded dragons (*Pogona vitticeps*), and a savannah monitor (*Varanus exanthematicus*)^12^. Cases of malignant iridophoromas were found in snakes^14,15^, in a dwarf bearded dragon (*Pogona henrylawsoni*)^16^ and in green iguana (*Iguana iguana*)^17^.

Xanthophoromas in reptiles have been also observed^15^. The reports of melanophoromas in reptiles indicate a visceral metastatic character of the tumour, whereas iridophoromas can occur with or without the visceral metastases^12^ and affecting only the skin. The aim of this study is to describe iridophoromas in *Eublepharis macularius* and to the best of the authors’ knowledge this is the first report of such kind of a tumour within *Eublepharis macularius*.

## Materials and Methods

### Ethical note

This study did not perform any experiments on animals. All performed examinations and samples collection were done during routine veterinary practice and did not require local ethics committee approval. All methods were performed in accordance with the relevant guidelines and regulations. Leopard geckos owner gave a permission for treatment and the use of samples to subsequent diagnostics.

### Materials

**Figure 2:**
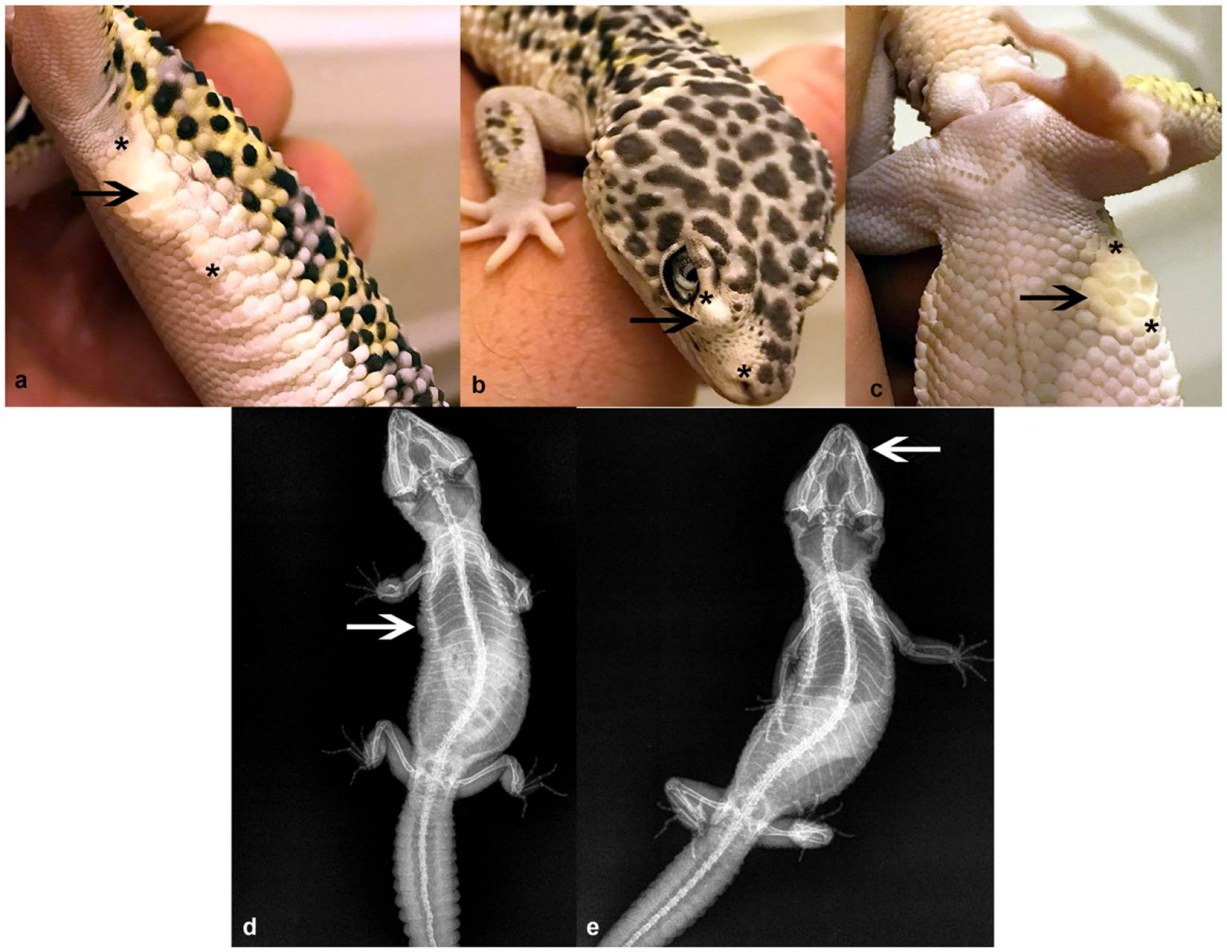
Example of the distribution of skin lesions. (**a–c**) Black arrows: from pale to white lesions. Black asterisks: point-to-point range of skin lesions. Radiograms of leopard geckos with skin lesions which resemble the typical soft tissue views (white arrows) (**d,e**).

In this report leopard geckos sourced from a private owner were examined in our clinic (Faculty of Veterinary Medicine, Wroclaw University of Environmental and Life Sciences) in relation to the presence of tumour-like skin lesions. Three geckos were selected for clinical examination: the male Snow Lemon Frost (weight 86 g, age 10 months), the male Hypo Lemon Frost (weight 55 g, age 10 months) and the female Eclipse (weight 57 g, age 3 years). All of the geckos were in good body condition, weight and size adequate to their age. According to the patient history we found that the Snow Lemon Frost and the Hypo Lemon Frost were related (via crossing different females with the same male), and that the Eclipse had a cross-fertilization with a Lemon Frost in its lineage. The animals were kept in a rack-system type of enclosure under a temperature gradient (26–30 °C), 12 hour day/night cycle and without UVB lightning. The animals were fed with mealworms supplemented with vitamin powder. All of the geckos were affected by nodular lesions of the skin (Fig. 2a–c), which emerged about two months earlier and affected 100% of leopard geckos of this colour morph from this owner. The lesions were painless, immobile and located on different parts of the body: eyelids, neck and both ventral and dorsal parts. The lesions had round or irregular shape varying from 0.5 to 2.0 cm in the longer diameter and ca 0.5 cm in the shorter diameter. The animals showed no other clinical symptoms.

### Methods

The diagnostic procedure included X-ray imaging (GIERTH HF 200, Germany) and ultrasonography (Supersonic Aixplorer^®^MultiWave™ Ultrasound, France), which were performed to check their effectiveness in the diagnosis of the lesions, the presence of putative metastases or any other concomitant internal pathologies. Several biopsies were taken from the nodular-like oval-shaped lesions with healthy skin margins from the necks or bodies of all three geckos. Not all of the skin lesions were qualified for an excision. Only the lesions located in a body region where about 0.5 cm of a healthy skin margin could be taken were chosen for a procedure (e.g. lesions located in a direct vicinity of an eyeball were excluded) (Fig. 2b). Sedation was applied using a standard protocol with 5% isoflurane (Piramal Healthcare, UK). The biopsy wounds were stitched with absorbable sutures (4–0 polyglycolide monofilament). Analgesic meloxicam was administered at a dose of 0.1 mg/kg. The samples were fixed in buffered 4% formaldehyde and routinely processed in paraffin. The 7 µm-thick cut sections were stained in haematoxylin and eosin (H&E), then analysed histopathologically in the light microscopy (Eclipse 80i, Nikon, Melville, NY, USA) and in the Nomarski contrast (Differential interference contrast, DIC) to provide evidence of the iridophorous character of the cells. After the surgical treatment the leopard geckos were given back to the owner.

### Ethics approval and consent to participate

This study does not perform any experiments on animals. All performed examinations and samples collection were done during routine veterinary practice and did not require local ethics committee approval.

## Results

X-ray analysis showed shading typical for soft tissues clearly visible as an enlarged contour of body and head soft tissues. None of the three geckos presented any abnormalities suggestive of metastases (Fig. 2d,e).

Ultrasound was used to examine several organs: the gallbladder (which presented normal with correct wall thickness, filled with clear bile), kidneys (correct shape and size), spleen (correct and homogeneous), liver parenchyma (homogeneous), liver vascular system (without abnormalities), stomach and intestines (normal, with food content). The ultrasound findings listed above apply to all the specimens.

**Figure 3:**
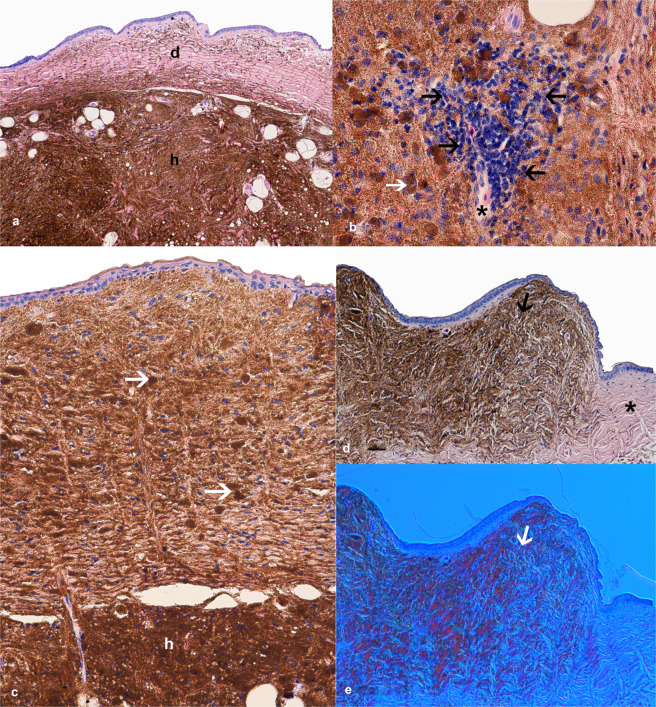
Histopathology of the leopard gecko skin iridophoroma. (**a–d)** H&E. (**e)** Nomarski contrast (DIC). (**a**) Large mass of iridophores in hypodermis (h) and scattered clusters of iridophores in dermis (d), regular collagen fibre arrangements were noted. (**b**) Infiltration of lymphohistiocytic cells (black arrows) around a skin capillary (black asterisk), atypical oval shaped iridophore with eccentrically located nucleus and brown cytoplasm (white arrow). (**c**) Tumour cells present both in the epidermis, dermis and hypodermis (h), round and spindle shaped cells filled with guanine crystals (white arrows), regular collagen fibre arrangement. (**d**) Overview of the affected skin with a tumour lesion (black arrow) and healthy margin without iridophores and other chromatophores (black asterisk). (**e**) Nomarski contrast used to confirm the presence of iridophores in the skin sections; note the characteristic change in colour of light-reflecting cells (white arrow).

Histopathological investigation revealed tumorous changes that consisted of iridophores, localised in the dermis and hypodermis of all the three geckos (Fig. 3). The cells contained considerable amounts of anisotropic crystalline material that caused characteristic polarization in the Nomarski contrast (DIC), (Fig. 3e). In all three individuals iridophores almost exclusively occupied the hypodermis, while in the dermis number of these cells varied between samples and individuals, that they cover from part (Fig. 3a) to almost whole field of view (Fig. 3c). Iridophores were generally chaotically distributed, locally in a vortex arrangement (Fig. 3a–c). The cells were spindle-shaped, with marked pigmentation and mostly without mitotic figures in a high power field (HPF). The cell nuclei were oval in shape, with mean diameters of 8 × 4 μm, but some of them revealed hyperpigmentation and irregular shape of the nucleus (*heteronucleosis*). Some iridophores presented atypical morphology: larger diameter, oval shape, eccentrically located nucleus and brown cytoplasm. In the dermis the cells were surrounded by connective tissue with a typical structure and arrangement of collagen fibres. The processes of iridophore-like cells spread among collagen fibres. However, in the hypodermis, connective tissues were scant and iridophore processes interwoven. This histopathological view indicated the malignant iridophoroma. These changes were accompanied by scant lymphohistiocytic cell infiltrates. The margins of the healthy skin did not reveal any presence of iridophores.

Although there are no widely accepted standards for a convalescence time after a skin surgery in geckos, it is reasonable to proceed veterinary check-ups twice: after a wound healing time (about a week), and after the very next moulting. Unfortunately, the owner of the affected leopard geckos did not agree to clinical control examinations. However, after 6 months he assured, that surgically removed skin lesions did not reveal recurrence, but new ones appeared in other body localizations. Furthermore, he did not observe any other abnormal symptoms like weight or appetite loss.

## Discussion

Leopard geckos are a very popular species that are kept in captivity. To date there is no information in a literature about iridophore-derived type of tumour in a wild-type or other leopard gecko colour morphs.

Histopathological analyses of the presented cases indicated that the tumorous-like lesions contained iridophore-like cells, which could not be found in the healthy skin margins. Light microscopy and DIC examination confirmed that the observed chromatophores were iridophores because of their light-reflecting character and the presence of anisotropic crystalline material in the cytoplasm what is characteristic for this cell type^14,18,19^. The morphology of the iridophores and infiltrative nature of the tumour indicates the malignant character of these changes. Surprisingly, our previous studies indicated that there is lack of iridophores in the skin of healthy animals of this species^8^. This phenomenon was also confirmed the present study by observation of healthy skin around the tumorous changes. The malignancy of the tumour-forming cells was determined pathomorphologically by the cells morphology, the infiltrative character of the changes that invade dermis and hypodermis^12^. Observed lymphohistiocytic cell infiltrations were small and scant and clinical observations revealed no signs such as inflammation, pain or any other discomfort in the patients. What more, the geckos arriving at our clinic in satisfying body condition displayed normal behaviour. The findings and the observations were not an indication for a euthanasia. Additionally, X-ray and ultrasonography revealed no signs of visceral metastases presence. According to our observations, surgical procedure is strongly recommended for operable lesions. The procedure is an effective method for a complete removal of the lesions, and no relapses have been observed so far. Nonetheless, it does not prevent the appearing of new ones in other body parts.

The presented cases are interesting for a few reasons. First, Heckers *et al*. (2012) suggested that chromatophoromas occur significantly more frequently in day-active reptiles like bearded dragons (*Pogona* sp.)^12^ than in nocturnal, cryptic species like leopard geckos. To the best of our knowledge this is the first study describing the iridophoroma in leopard geckos, a popular species bred in captivity. Second, our findings indicate that a selective inbreeding influences the disease incidence. Additionally, it cannot be excluded that the cross-fertilization of morphs with the Lemon Frost may be associated with an increased incidence of iridophoroma in next generations (e.g. the Eclipse patient). Our findings are the first observation of iridophoromas in leopard geckos and further research should consider statistical analysis of different colour morphs crossing with Lemon Frost to fully establish which crosses yield offspring with iridophoromas.

In view of a putative connection between iridophoromas and the genome, further breeding of the Lemon Frost leopard gecko line is not recommended until the pathogenesis of the lesions will be fully recognised and described.

## Data Availability

All data generated or analysed during this study are included in this published article.
