# Author Correction: Iridophoroma associated with the Lemon Frost colour morph of the leopard gecko (*Eublepharis macularius*)

**DOI:** 10.1038/s41598-021-87009-0

**Published:** 2021-03-25

**Authors:** Paweł Szydłowski, Jan Paweł Madej, Magdalena Duda, Janusz A. Madej, Agnieszka Sikorska-Kopyłowicz, Anna Chełmońska-Soyta, Lucyna Ilnicka, Przemysław Duda

**Affiliations:** 1 Department of Immunology, Pathophysiology and Veterinary Preventive Medicine, Faculty of Veterinary Medicine, Wroclaw University of Environmental and Life Sciences, Norwida 31, 50-375 Wroclaw, Poland; 2 Department of Histology and Embryology, Faculty of Veterinary Medicine, Wroclaw University of Environmental and Life Sciences, Norwida 25, 50-375 Wroclaw, Poland; 3 Department of Internal Diseases and Clinic of Diseases of Horses, Dogs and Cats, Faculty of Veterinary Medicine, Wroclaw University of Environmental and Life Sciences, pl. Grunwaldzki 47, 50-366 Wroclaw, Poland; 4 Department of Pathology, Faculty of Veterinary Medicine, Wroclaw University of Environmental and Life Sciences, Norwida 31, 50-375 Wroclaw, Poland; 5 Department of Epizootiology and Clinic of Bird and Exotic Animals, Faculty of Veterinary Medicine, Wroclaw University of Environmental and Life Sciences, pl. Grunwaldzki 45, 50-366 Wroclaw, Poland; 6 Department of Molecular Physiology and Neurobiology, University of Wroclaw, Sienkiewicza 21, 50-335 Wroclaw, Poland

Correction to: *Scientific Reports* 10.1038/s41598-020-62828-9, published online 31 March 2020

The Funding section in this Article was omitted. The Funding section should read:

‘The costs of publication were financed under the Leading Research Groups support project from the subsidy increased for the period 2020–2025 in the amount of 2% of the subsidy referred to Art. 387 (3) of the Law of 20 July 2018 on Higher Education and Science, obtained in 2019.’

